# Concentric rings with k-t acceleration enables rapid and effective fat-water-separated cardiac cine MRI at 3 T

**DOI:** 10.1186/1532-429X-15-S1-P52

**Published:** 2013-01-30

**Authors:** Holden H Wu, Taehoon Shin, Dwight G Nishimura, Michael V McConnell

**Affiliations:** 1 Cardiovascular Medicine, Stanford University, Stanford, CA, USA; 2 Electrical Engineering, Stanford University, Stanford, CA, USA

## Background

Cine imaging is the core technique for cardiac MRI studies. Often times, it is also essential to have information identifying fat signal in the heart to characterize masses, detect fatty infiltration and ARVD, discern fat from fibrosis in LGE imaging, and improve visualization of the pericardium. However, such information traditionally requires additional scans that obtain fat-suppressed images for comparison. This prolongs the examination time, is susceptible to mis-registration between the fat-suppressed and fat-visible images, and does not directly utilize positive contrast from fat. In this work, we present an integrated cardiac MRI technique that produces co-registered cines of both water and fat signal from a rapid single-breath-hold scan.

## Methods

**Figure 1:**
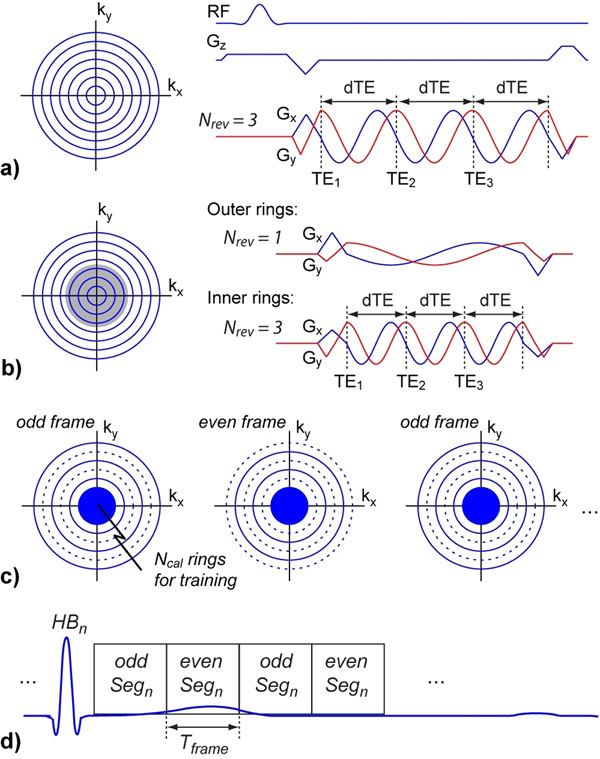
Concentric rings. **(a)** 2D k-space is sampled with uniformly spaced concentric rings, where gradients are designed for the outermost ring and then scaled down to acquire one ring after each RF pulse. A time-efficient retracing design is used to acquire each ring through multiple revolutions. Each revolution captures the image at a different effective TE_r_ (separation dTE) to enable fat-water separation. **(b)** Extension to a two-band design where only the inner half of k-space (shaded) is acquired with retracing. This can increase gradient power for the inner rings and decrease dTE. **(c)** Time-interleaved k-t undersampling for R = 2. Rings denoted by dotted lines are omitted, while a central region of *N_cal_* rings is always acquired for training. **(d)** The desired sets of rings for each frame are acquired over multiple heartbeats (HB) with interleaved segmentation. Temporal resolution is *T_frame_*.

A concentric rings readout trajectory with multiple revolutions per TR is used to simultaneously encode 2D spatial and 1D chemical shift information (Fig. 1a). Gradients are designed for the outermost ring and scaled down to acquire all rings. Compared to 2DFT, the concentric rings require half the number of excitations, thus can achieve up to 2-fold reduction in scan time. Similar to multi-echo 2DFT, each revolution can be reconstructed as a separate source image with effective echo time TE_r_ (r = 1 to N_rev_). Dixon-type fat-water separation can then be performed using these source images. The multi-revolution acquisition ensures that the source images, and hence the calculated fat and water images, are inherently co-registered. The basic concentric rings design can be extended to a two-band design (Fig. 1b) where only the inner rings are retraced. This helps to reduce echo separation dTE and accommodate the greater fat-water chemical shift and field inhomogeneity at 3 T. To further reduce scan time, the concentric rings are combined with k-t BLAST. Time-interleaved undersampling is implemented (R = 2 in Fig. 1c) and the undersampled k-space for each cine frame is acquired as interleaved segments over multiple heartbeats (Fig. 1d).

## Results

**Figure 2:**
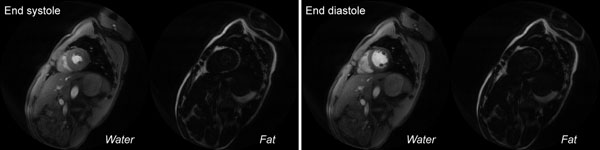
3 T short-axis cardiac cine results. A two-band concentric rings design was implemented, with the inner 65 (of 130) rings acquired over N_rev_ = 3 revolutions. Prospective k-t undersampling was performed with *N_cal_* = 19 and R = 3. Scan time was 14 heartbeats (10 sec at 80 bpm) and *T_frame_* was 24.8 ms.

The concentric rings were implemented in a breath-held 2D GRE sequence with prospective cardiac triggering. A set of 130 rings encoded an FOV of 40x40 cm^2^ with spatial resolution of 1.54x1.54 mm^2^. The inner 65 rings were acquired with N_rev_ = 3 (dTE = 1.024 ms). Prospective k-t undersampling was performed, with N_cal_ = 19 and R = 3. Sequence parameters were 8-mm slice, TE_1_ / TR / FA = 1.2 ms / 6.2 ms / 15 deg, 4 rings/segment (T_frame_ = 24.8 ms/frame), and 24 cardiac phases. Total scan time was 10 sec (heart rate of 80 bpm). Fat-water-separated cine frames obtained from a healthy volunteer at 3 T are shown in Fig. 2. Uniform fat-water separation is achieved over the entire FOV.

## Conclusions

By combining concentric rings with k-t BLAST, co-registered water and fat cines can be obtained at 3 T with 25-ms temporal resolution within a single 10-sec breath-hold scan. Cardiac function and fat distribution can be simultaneously visualized for a range of applications.

## Funding

NIBIB T32 EB009035; NHLBI R01 HL039297; AHA 11POST7420014; GE Healthcare

